# Spontaneous Splenic Rupture in Melanoma

**DOI:** 10.1155/2014/865453

**Published:** 2014-03-26

**Authors:** Hadi Mirfazaelian, Ahmad Oryan, Aida Davari, Khosrow Daneshbod, Yahya Daneshbod

**Affiliations:** ^1^Department of Emergency Medicine, Tehran University of Medical Sciences, Tehran, Iran; ^2^Department of Pathology, School of Veterinary Medicine, Shiraz University, Shiraz, Iran; ^3^Dr. Daneshbod Pathology Laboratory, Department of Pathology, Shiraz, Iran

## Abstract

Spontaneous rupture of spleen due to malignant melanoma is a rare situation, with only a few case reports in the literature. This study reports a previously healthy, 30-year-old man who came with chief complaint of acute abdominal pain to emergency room. On physical examination, abdominal tenderness and guarding were detected to be coincident with hypotension. Ultrasonography revealed mild splenomegaly with moderate free fluid in abdominopelvic cavity. Considering acute abdominal pain and hemodynamic instability, he underwent splenectomy with splenic rupture as the source of bleeding. Histologic examination showed diffuse infiltration by tumor. Immunohistochemical study (positive for S100, HMB45, and vimentin and negative for CK, CD10, CK20, CK7, CD30, LCA, EMA, and chromogranin) confirmed metastatic malignant melanoma. On further questioning, there was a past history of a nasal dark skin lesion which was removed two years ago with no pathologic examination. Spontaneous (nontraumatic) rupture of spleen is an uncommon situation and it happens very rarely due to neoplastic metastasis. Metastasis of malignant melanoma is one of the rare causes of the spontaneous rupture of spleen.

## 1. Introduction

Splenomegaly is defined when the length of spleen of a mature person is more than 12 cm. Symptoms of splenomegaly may include abdominal pain, chest pain, back pain, early satiety, anemia, and palpable left upper quadrant abdominal mass or splenic rub. It can be detected on physical examination by Castell's sign or Traube's space but an ultrasound can be used to confirm the diagnosis. Causes of splenomegaly are spherocytosis, thalassemia, hemoglobinopathy, nutritional anemia, early sickle cell anemia, immune hyperplasia, mononucleosis, AIDS, viral hepatitis, subacute bacterial endocarditis, lymphoma, and melanoma metastasis to the spleen. According to a report, the lung cancer, cutaneous malignant melanoma, and breast cancer are the most frequent sources of splenic metastases, respectively [1].

Melanoma, an important splenic metastatic tumor, is a tumor of melanocytes which are found predominantly not only in skin but also in the bowel and the eyes. It is one of the less common types of skin cancers but causes the majority (75%) of skin cancer-related deaths. Melanocytes are normally present in skin, being responsible for production of the dark pigment melanin. According to some of the previous reports, melanoma causes splenomegaly and in rare instances spontaneous splenic rupture [2, 3].

There have been several reported cases of splenic rupture in leukemia [2, 4]. Spontaneous splenic rupture as the first presentation of metastatic melanoma to the spleen is very rare [3, 5]. Despite the high incidence of splenic metastases in metastatic melanoma, there have been very few cases of spontaneous splenic rupture reported in the literature [2]. However, there are several reports regarding splenic metastatic melanoma.

## 2. Case Report

A 30-year-old man presented with severe abdominal pain and spontaneous intra-abdominal bleeding. Diagnostic imaging failed to prove another site of melanoma, and no history of melanoma or cutaneous lesion was reported by the patient. Abdominal imaging showed splenomegaly. Liver was normal in size with no sign of space occupying lesion or bile duct dilatation. Gall bladder was well distended with no sign of stone or wall thickening. Moderate to severe free fluid was noted in abdominopelvic cavity. Kidneys, ureters, urinary bladder, prostate, and seminal vesicles were normal.

**Figure 1:**
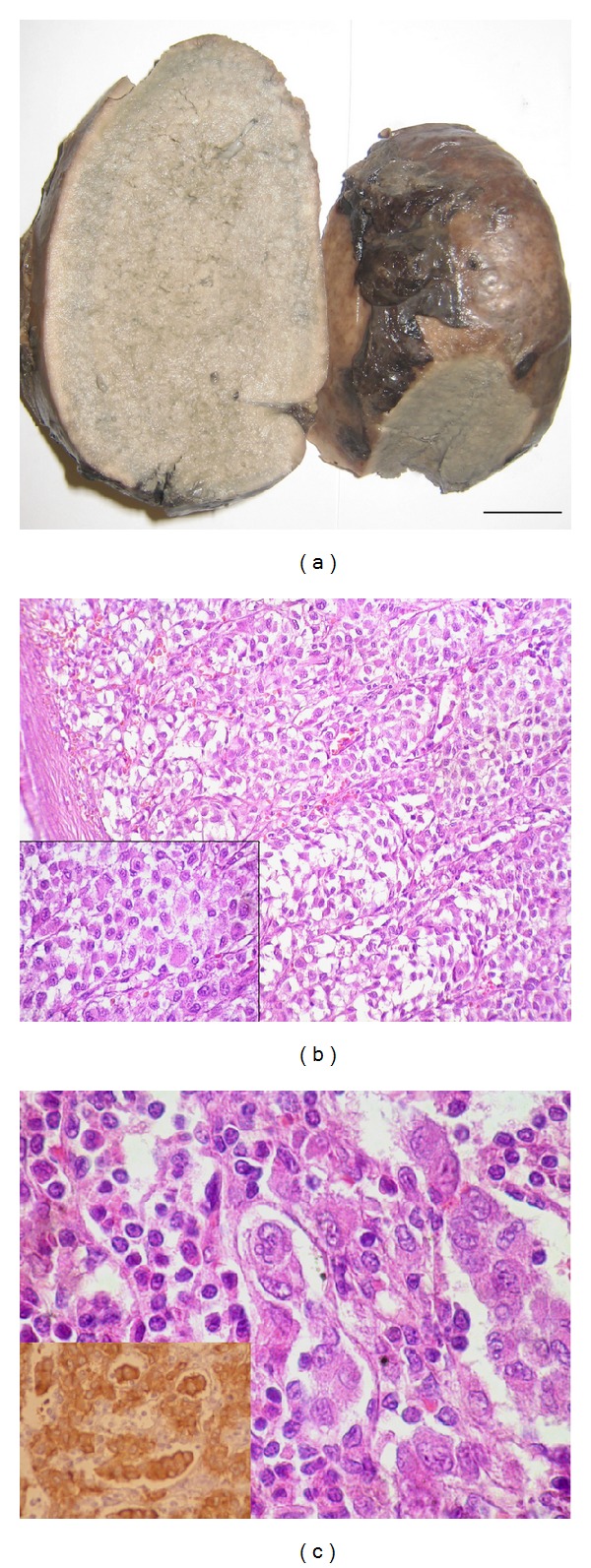
(a) Gross pathology: capsular laceration and hemorrhage and cut sections show diffuse involvement of spleen by creamy homogenous tumoral tissue. (b) Diffuse involvement of spleen by malignant melanoma cells showing fascicular and trabecular pattern (Hematoxylin and Eosin stain, scale bar = 100 *μ*m). Inset shows rhabdoid type tumor cells (Hematoxylin and Eosin stain, scale bar = 50 *μ*m). (c) High power shows tumoral cells in red pulp and sinusoids (Hematoxylin and Eosin stain, scale bar = 25 *μ*m). Inset shows positive immunoperoxidase for HMB-45 in tumoral cells (immunoperoxidase, scale bar = 25 *μ*m).

Splenectomy was performed. After fixation in 10% neutral buffered formalin, the spleen samples were washed, dehydrated, cleared, embedded in paraffin wax, sectioned at 4-5 *μ*m, stained, and examined by a light microscope. The ruptured and fragmented spleen's dimensions were 14 × 10 × 6 cm. On gross pathology, the capsular surface showed sites of laceration and hemorrhages and on cut surface there was diffuse creamy homogenous splenic involvement (Figure 1(a)). Histologic examination showed diffuse infiltration by tumor cells in fascicular and trabecular pattern (Figure 1(b)) with some rhabdoid-like cells (Figure 1(b) inset) occupying mainly red pulp and sinusoids (Figure 1(c)). These cells were found out to be of melanocytic origin and melanoma was confirmed with immunohistochemical study (positive for S100, HMB45 (Figure 1(c) inset), melan A, and Vimentin and negative for CK, CD10, CK20, CK7, CD30, LCA, EMA, and Chromogranin).

## 3. Discussion

Postmortem studies in patients with cancer show the incidence of splenic metastases to be 2.3–12.9% [6]. Almost all common tumors have been reported to some stages to give rise to splenic secondaries, the most frequent being lung, breast, malignant melanoma, and ovary. Berge's large postmortem series found splenic metastases in 7.1% of cases with carcinoma, with the spleen being the 10th most frequent site of metastasis [6]. Splenic secondaries were present in 12% of patients with breast cancer. Splenic metastases are generally seen in patients with advanced disease, many having metastases at several other sites. Metastatic disease in the spleen is often asymptomatic but may present with discomfort in the left upper quadrant and symptoms related to pressure on other organs, for example, early satiety and dyspnea. There may also be features of disseminated systemic disease, such as cachexia and haematological abnormalities as a result of hypersplenism, particularly where there is diffuse parenchymal involvement [7]. Splenic metastases from most types of tumor are rare, but they occur more frequently in patients with breast cancer, lung cancer, and melanoma.

Hess et al. [5] reported a case of metastatic melanoma with atypical invasion to the spleen. Kyser et al. [8] reported a patient with melanoma and metastases to the spleen and lung that resulted in severe painful splenomegaly. Richter et al. [9] reported spontaneous splenic rupture in a metastatic malignant melanoma.

The majority of splenic metastases from melanoma are identified at autopsy. In life, they are commonly part of a widespread, end stage disease [10].

Laparoscopic splenectomy is the preferred surgical approach for benign hematologic disorders when elective removal of the spleen is indicated. Technical success, minimal morbidity, reduced disability, and high patient acceptance have justified application of the laparoscopic splenectomy. Laparoscopic splenectomy can be performed, with good results, on patients with a variety of malignant hematologic disorders as well. The laparoscopic approach is a safe and effective treatment for splenic melanoma metastases [11].

The most common sources of splenic metastases are breast, lung, colorectal, and ovarian carcinoma and melanoma. Fine-needle aspiration and percutaneous biopsy of splenic lesions are useful in establishing the correct diagnosis in most cases. According to recent advances in the knowledge of the metastatic process, it seems likely that late occurrence of solitary splenic metastases might develop from early blood-borne micrometastasis within the spleen after a period of clinical latency [12].

Determination of the primary versus secondary lesion may be difficult in cases without a definitive medical history of a cutaneous primary or long-term clinical follow-up. In our case, there was no history of a current or prior melanotic skin lesion, and there was no other organ involvement on computed tomography imaging or bone scintigraphy scan.

Despite the low frequency of metastatic tumors in the spleen (4%), these are more frequently encountered in autopsies of patients with melanoma (36%) [13].

The diagnosis of metastatic melanoma in the spleen is rare, although this patient presented splenic metastases at the time of diagnosis. Marked splenomegaly is not common, probably because the average size of the nodules is 1.5 cm [13].

These metastases result from either lymphatic or vascular dissemination [13]. The factors responsible for the death of the present patient were probably hemorrhagic and metabolic complications.

The incidence of and mortality from melanoma are increasing and no effective treatment for the disseminated disease exists. Programs for prevention and early detection of melanoma are therefore warranted [14].

Spontaneous (nontraumatic) rupture of spleen as an uncommon and rare situation has happened due to neoplastic metastasis. Malignant melanoma of spleen could be considered as rare cause of acute abdominal pain and nontraumatic spontaneous splenic rupture.
